# Vaginal Practices among Women at High Risk of HIV Infection in Uganda and Tanzania: Recorded Behaviour from a Daily Pictorial Diary

**DOI:** 10.1371/journal.pone.0059085

**Published:** 2013-03-26

**Authors:** Suzanna C. Francis, Kathy Baisley, Shelley S. Lees, Bahati Andrew, Flavia Zalwango, Janet Seeley, Judith Vandepitte, Trong T. Ao, Janneke van de Wijgert, Deborah Watson-Jones, Saidi Kapiga, Heiner Grosskurth, Richard J. Hayes

**Affiliations:** 1 Department of Infectious Disease Epidemiology, London School of Hygiene and Tropical Medicine, London, United Kingdom; 2 Mwanza Intervention Trials Unit, National Institute for Medical Research Mwanza Centre, Mwanza, Tanzania; 3 MRC/UVRI Uganda Research Unit on AIDS, Uganda Virus Research Institute (UVRI), Entebbe, Uganda; 4 School of International Development, University of East Anglia, Norwich, United Kingdom; 5 Institute of Infection and Global Health, University of Liverpool, Liverpool, United Kingdom; 6 Department of Clinical Research, London School of Hygiene and Tropical Medicine, London, United Kingdom; UCL Institute of Child Health, University College London, United Kingdom

## Abstract

**Background:**

Intravaginal practices (IVP) are highly prevalent in sub-Saharan African and have been implicated as risk factors for HIV acquisition. However, types of IVP vary between populations, and detailed information on IVP among women at risk for HIV in different populations is needed. We investigated IVP among women who practice transactional sex in two populations: semi-urban, facility workers in Tanzania who engage in opportunistic sex work; and urban, self-identified sex workers and bar workers in Uganda. The aim of the study was to describe and compare IVP using a daily pictorial diary.

**Methodology/Principal Findings:**

Two hundred women were recruited from a HIV prevention intervention feasibility study in Kampala, Uganda and in North-West Tanzania. Women were given diaries to record IVP daily for six weeks. Baseline data showed that Ugandan participants had more lifetime partners and transactional sex than Tanzanian participants. Results from the diary showed that 96% of Tanzanian participants and 100% of Ugandan participants reported intravaginal cleansing during the six week study period. The most common types of cleansing were with water only or water and soap. In both countries, intravaginal insertion (e.g. with herbs) was less common than cleansing, but insertion was practiced by more participants in Uganda (46%) than in Tanzania (10%). In Uganda, participants also reported more frequent sex, and more insertion related to sex. In both populations, cleansing was more often reported on days with reported sex and during menstruation, and in Uganda, when participants experienced vaginal discomfort. Participants were more likely to cleanse after sex if they reported no condom use.

**Conclusions:**

While intravaginal cleansing was commonly practiced in both cohorts, there was higher frequency of cleansing and insertion in Uganda. Differences in IVP were likely to reflect differences in sexual behaviour between populations, and may warrant different approaches to interventions targeting IVP. Vaginal practices among women at high risk in Uganda and Tanzania: recorded behaviour from a daily pictorial diary.

## Introduction

There is evidence that some types of intravaginal practices (IVP) are a risk factor for bacterial vaginosis and HIV infection among women in sub-Saharan Africa [1], [2]. IVP include any practice in which a substance (eg. water, soap, antiseptic, herbs) is applied inside the vagina by different application methods (eg. finger washing, cloth wiping). The recent WHO Gender, Sexuality and Vaginal Practices (GSVP) study on vaginal practices classified IVP into two types: intravaginal cleansing and intravaginal insertion (i.e. placing something inside the vagina, such as herbs) [3]. Both types of IVP are hypothesised to disrupt the normal protective cervicovaginal microenvironment, thus increasing susceptibility to bacterial vaginosis and HIV [3]–[8]. There is also concern that IVP may interfere with the testing of candidate vaginal microbicides for HIV prevention or the effectiveness of an efficacious vaginal microbicide [3], [9], [10]. Although IVP are highly prevalent in many areas of sub-Saharan Africa, substances and methods of application vary from region to region [9], [11]; consequently, it is important to obtain detailed information on IVP in different study populations.

While most studies use retrospective face-to-face interviews (FTFI) to obtain data on IVP, FTFI can be subject to recall and social desirability bias, and may oversimplify IVP use by asking participants to summarize their practices over time. Diaries have been used to collect sensitive behavioural data among women at high risk for HIV in sub-Saharan Africa since the late 1990 s [12]–[15]. We carried out a study using a daily, self-completed, vaginal practices diary to obtain information on IVP frequency and characteristics, and in-depth interviews (IDI) to obtain further detail on IVP and to contextualise practices within local norms. The aim of this study was to describe and compare IVP within two study populations of women at increased risk for HIV in Tanzania and Uganda. This paper reports the findings from the diaries, drawing on the IDI data to aid with the interpretation of results. The full results from IDI and other qualitative data will be presented in detail in a subsequent paper.

## Materials and Methods

### Ethics Statement

This study was approved by the ethics committees of the London School of Hygiene and Tropical Medicine, the Tanzanian National Institute for Medical Research, the Science and Ethics Committee of the Uganda Virus Research Institute, and the Ugandan National Council for Science and Technology. Informed consent was obtained in writing or by fingerprint from all participants prior to enrolment.

### Study design and participants

The Diary Study was carried out in study sites in two areas in East Africa: Shinyanga and Geita Regions in North-West Tanzania and in Kampala City, Uganda. In both countries, participants were currently enrolled in large, observational cohorts of women at increased risk of HIV. In Tanzania, the Diary Study was nested within an occupational cohort study in three towns located near gold and diamond mines. All women enrolled in this cohort were employed in bars, guesthouses and other food and recreational facilities. Previous studies have shown that such female facility workers are at high risk for HIV infection [12], [16], [17], as many engage in opportunistic transactional sex. The cohort enrolled 970 HIV negative, non-pregnant women and followed them every three months for one year. Baseline findings have been reported elsewhere [18]. For the Diary Study, 100 women from two of the three study sites were randomly selected from the 663 enrolled women in the main cohort study. Consenting participants were asked to fill out the diary every day for six weeks (42 days), and to participate in an IDI at the end of this period. Participants were visited by a research assistant on the second day of the study to check on diary understanding and then at weekly intervals to collect completed diaries, answer questions, and issue new diaries. All participants in the Tanzanian Diary Study were seen during the six weeks between enrolment and their first three-month follow-up visit.

In Uganda, the Diary Study was nested within a cohort of women in Kampala, Uganda. Participants in the cohort were either self-identified female sex workers (FSW) or employed in city entertainment facilities such as bars, nightclubs, and lodges. Between April 2008 and April 2009, 1027 women (645 HIV negative and 382 HIV positive) were enrolled and followed every three months in an on-going cohort study. Baseline findings have been reported elsewhere [19]. Between July and September 2009, 100 women were enrolled into the Diary Study by selecting every fourth participant at any follow up visit: 9 were enrolled at their 3 month clinic visit, and the remainder distributed approximately equally between their 6–15 month visits. Both Diary Study cohorts used a similar protocol, and the diary design was identical except for the drawings which were adapted to be locally appropriate. Women who were pregnant or currently breastfeeding were excluded from the diary study so that IVP during menstruation could be documented.

### Vaginal practices diary

**Figure 1:**
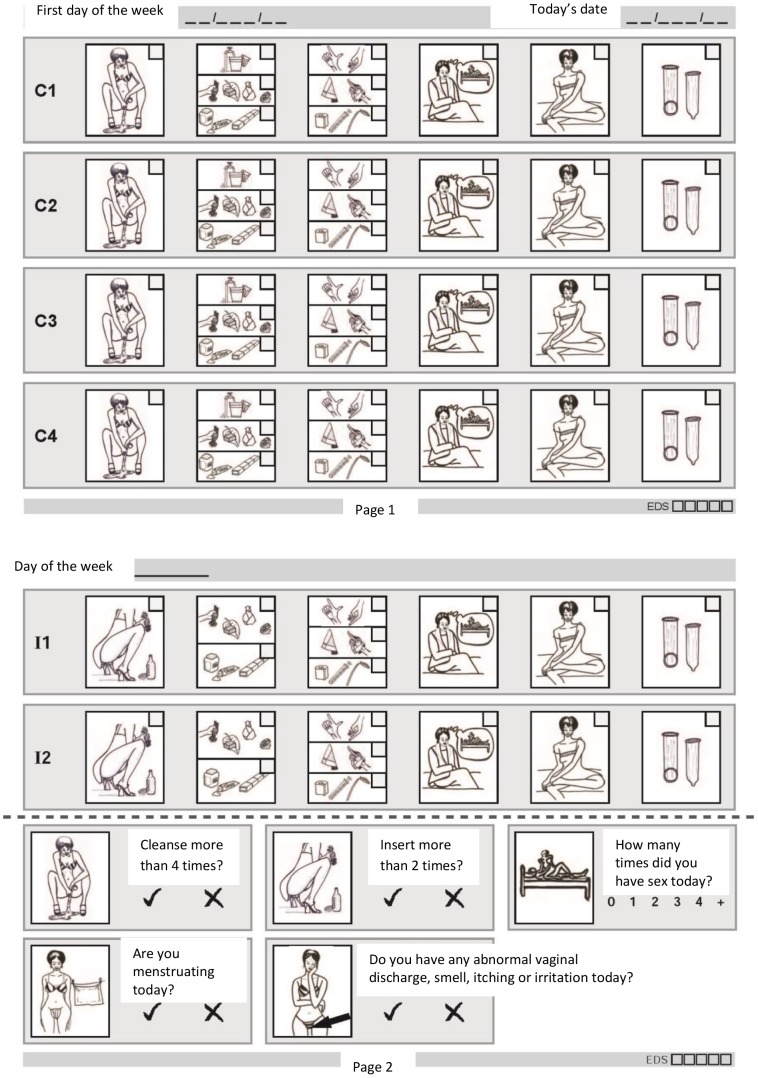
The vaginal practice diary in Tanzania. There are two pages for each day. On page one there are four rows to report individual acts of cleansing. If a participant ticks a cleansing box then the adjoining horizontal rows must be ticked or crossed to describe that act of cleansing. One page two there are two insertion rows to report individual acts of insertion, with adjoining horizontal rows to describe each act. There are five boxes at the bottom of page two in which a participant reports more than 4 acts of cleansing, more than two acts of insertion, menstruation, vaginal discomfort, and number of times she had sex on that day. The Ugandan diary is identical to the Tanzania diary except the pictures were adapted by a local Ugandan artist.

A pictorial vaginal practices diary was developed for this study by adapting a published coital diary used for a multi-site microbicide clinical trial in Tanzania (Mwanza), Uganda (Masaka) and 4 other study sites [12], [14]. Development of the vaginal practice diary has been described elsewhere [20]. In brief, focus group discussions were held with the study populations and a local artist in order to adapt the diary pictures, and several pilots (three in Tanzania and one in Uganda) were carried out until the design was optimized. The final, modified diary is shown in Figure 1. In the diary, up to four cleansing acts and two insertion acts can be described each day, and substances used for cleansing and insertion are summarized as either commercial or traditional substances. Participants were told that commercial products would be made in factories and sold in grocery stores, and traditional substances were either natural substances, or locally made substances sold in local markets or by traditional healers. In the diary, women were also asked if they cleansed more than 4 times, or inserted more than 2 times, and how many times they had sex each day. A semi-structured IDI obtained data on practice initiation, the exact type of products used, motivations for using the practice, physical results and harmful effects from using the practice, and if the participant's partner was aware of the practice. Participants were also interviewed about their work, family life, sexual relationships, hygiene during menstruation, and general bathing habits.

### Statistical analysis

Main study and diary data were double entered and verified at the study site and analysed using Stata, version 11 (StataCorp, College Station, Texas, USA). Within each Diary Study cohort, we compared baseline characteristics of the Diary Study participants with those of the main cohort, to assess whether the sub-sample was representative of the cohort. In addition, we compared the baseline characteristics of the two Diary Study cohorts, using Chi-squared and Wilcoxon rank-sum tests as appropriate.

Cleansing and insertion data were summarised using frequency counts, proportions, means and medians. The analysis was restricted to women who completed all six diaries. Cleansing data were summarised among all participants; however, since insertion was relatively uncommon, insertion data were summarised only among participants who reported insertion at least once during the study. Data were summarised on a per-woman and per act basis. The diary was designed to capture detailed information on only 4 cleansing acts and 2 insertion acts per day; therefore, when calculating the total number of cleansing or insertion acts, participants were assumed to have cleansed 5 times or inserted 3 times, on days when they reported >4 cleansing or >2 insertion acts. However, since detailed information was not gathered on these additional acts, they could not be described further.

For each woman, cleansing frequency was calculated as the total number of cleansing acts recorded in the diary divided by the total number of days with diary data. In the per-woman analysis, substance used for cleansing was categorised as “water only,” “traditional” if a traditional substance was used at least once during the six week study, or “commercial” if a commercial substance was used at least once. Application method for cleansing was categorised as “fingers only,” or “cloth” if a cloth was used at least once during the study period, or “other” if another applicator was used at least once.

When summarising the frequency of sex-related cleansing or sex-related insertion as a proportion of sex acts, the analysis was restricted to days when women reported ≤4 cleansing acts, or ≤2 insertion acts, respectively. This ensured that all sex-related IVP on that day would be captured by the diary.

Within each Diary Study cohort, the Wilcoxon signed-rank test for paired data was used to assess whether the frequency of cleansing or insertion differed between days when women reported or did not report sex acts, menstruation or vaginal discomfort. Vaginal discomfort was defined as self-reported vaginal itching, burning, discharge or smell.

In the per-act analysis, to investigate the relationship between condom use and the timing of sex-related IVP, we compared the frequency of condom use when cleansing or insertion was performed before sex, with that when it was performed after sex. On days when women reported both before- and after-sex IVP on the same diary page, the design of the diary did not allow us to assess if the IVP related to two separate sex acts, or a single one. Therefore, as a sensitivity analysis, we compared condom use and the timing of IVP on days when women reported only before-sex IVP or only after-sex IVP.

## Results

### Screening, enrolment and follow up

In Tanzania, 103 women were screened to participate in the Diary Study; two were excluded because they were breastfeeding and 101 were eligible for enrolment, of whom all but one joined the study (99%). In Tanzania, 92 women returned at least one diary, and 82 returned all six diaries, providing 492 of 600 (82.0%) women-weeks of follow-up. Participants who completed all six diaries were more likely to be older, employed for longer, married, had fewer life-time partners, older age of first sex and less transactional sex (data not shown).

In Uganda, 133 women were screened to participate in the Diary Study; 32 were excluded because they were pregnant or breastfeeding and 101 were eligible for enrolment, all of whom joined the study. In Uganda, 100 returned at least one diary and 99 returned all six diaries, providing 594 of 606 (98.0%) women-weeks of follow-up.

### Comparing each Diary Study cohort to the main cohort in each country

Although each site used a different method to select the sub-sample of 100 participants, there were few baseline differences between the sub-sample and the main cohort: in Tanzania, women in the Diary Study were more likely to engage in transactional sex (p = 0.07, Table 1), and more likely to report consistent condom use (p = 0.01) in the last three months than those in the main cohort; and in Uganda, women in the Diary study were more likely to be HIV positive (p = 0.02). Note that eligibility for the Tanzanian cohort was based on HIV seronegativity; therefore all Tanzanian participants were HIV negative at enrolment.

**Table 1:** Comparison of the Tanzanian and Ugandan cohorts and the sub-samples of women enrolled for the Diary Study.

	Tanzanianmain cohort(n = 970)	TanzanianDiary Study participants(n = 100)	Ugandanmain cohort(N = 1027)	UgandanDiary Study participants(n = 100)	Comparison of Diary Study Participants by country
**Age (years)**		p = 0.38^a^		p = 0.27^a^	p = 0.04^b^
<25	395 (40.7%)	43 (43.0%)	412 (40.1%)	32 (32.0%)	
25-29	246 (25.4%)	23 (23.0%)	337 (32.8%)	34 (34.0%)	
30-34	150 (15.5%)	11 (11.0%)	168 (16.4%)	20 (20.0%)	
35+	179 (18.5%)	23 (23.0%)	110 (10.7%)	14 (14.0%)	
**Employment**		p = 0.50		p = 0.26	<0.01
Female sex worker	0 (0%)	0 (0%)	699 (68.1%)	61 (61%)	
Bar worker	197 (20.4%)	23 (23.0%)	253 (24.6%)	31 (31%)	
Other facility worker	769 (79.6%)	77 (77.0%)	75 (7.3%)	8 (8.0%)	
**Time employed**		p = 0.52		p = 0.25	0.64
≤ 1 year	258 (26.6%)	22 (22.0%)	242 (24.6%)	23 (25.8%)	
1 to 3 years	455 (46.9%)	51 (51.0%)	508 (51.6%)	43 (44.3%)	
> 3 years	257 (26.5%)	27 (27.0%)	235 (23.8%)	29 (29.9%)	
**Highest level of education**		p = 0.10		p = 0.51	<0.01
Never went to school	100 (10.3%)	8 (8.0%)	85 (8.8%)	8 (8.0%)	
Primary incomplete	198 (20.4%)	19 (19.0%)	418 (40.7%)	42 (42.0%)	
Primary complete	550 (56.7%)	53 (53.0%)	151 (14.7%)	19 (19.0%)	
Entered secondary (Complete/ Incomplete)	121 (12.5%)	20 (20.0%)	373 (36.3%)	31 (31.0%)	
**Marital status**		p = 0.14		p = 0.37	<0.01
Married	204 (21.0%)	13 (13.0%)	83 (8.1%)	5 (5.0%)	
Separated/ divorced	452 (46.6%)	47 (47.0%)	656 (63.9%)	70 (70.0%)	
Widowed	31 (3.2%)	4 (4.0%)	59 (5.7%)	7 (7.0%)	
Single	283 (29.2%)	36 (36.0%)	229 (22.3%)	18 (18.0%)	
**Religion**		p = 0.88		p = 0.35	0.74
Christian	736 (76.6%)	76 (76%)	760 (74.0%)	78 (78.0%)	
Muslim	225 (23.4%)	24 (24%)	265 (25.8%)	22 (22.0%)	
**Total life-time partners**		p = 0.40		p = 0.75	<0.01
0 – 4	394 (40.6%)	36 (36.0%)	47 (4.6%)	3 (3.0%)	
5 – 9	210 (21.7%)	17 (21.7%)	82 (8.0%)	9 (9.0%)	
10 – 19	100 (10.3%)	11 (11.0%)	75 (7.3%)	10 (10.0%)	
20 – 49	43 (4.4%)	6 (6.0%)	80 (7.8%)	8 (8.0%)	
50+	26 (2.7%)	4 (4.0%)	63 (6.1%)	4 (4.0%)	
Don’t remember	193 (19.9%)	26 (26.0%)	680 (66.3%)	66 (66.0%)	
**Age of first sex**		p = 0.12		p = 0.23	<0.01
14 or younger	123 (12.7%)	9 (9.0%)	355 (34.6%)	40 (40.0%)	
15-16	314 (32.4%)	33 (33.0%)	378 (36.8%)	28 (28.0%)	
17-18	314 (32.4%)	36 (36.0%)	209 (20.4%)	20 (20%)	
19 or older	141 (14.5%)	9 (9.0%)	50 (4.9%)	8 (8.0%)	
Unknown	78 (8.0%)	13 (13.0%)	35 (3.4%)	4 (4.0%)	
**Condom use in the past 3 monthsc**		p = 0.01		p = 0.45	0.02
Consistent	503 (55.3%)	64 (67.4%)	846 (84.4%)	81 (81.8%)	
Inconsistent	406 (44.7%)	31 (32.6%)	156 (15.6%)	18 (18.2%)	
**Transactional sex in the past 3 months**		p = 0.07		p = 0.33	<0.01
No	601 (62.2%)	50 (54.0%)	42 (4.2%)	6 (6.1%)	
Yes	365 (37.8)	46 (46.0%)	960 (95.8%)	93 (93.9%)	
**HIV Status at enrolmentd**		N/A		p = 0.02	<0.01
Negative	970 (100%)	100 (100%)	645 (62.8%)	52 (52.0%)	
Positive	0 (0%)^c^	0 (0%)	382 (37.2%)	48 (48.0%)	

**Legend:** a =  p-values comparing women participating in Diary Study with those in the main cohort who did not participate in the Diary Study, within each site by chi square; b =  p-values comparing the Diary participants between the two sites by chi square; c  =  Consistent condom use defined as reported condom use in the past three month was Always/Most of the time; d  =  Eligibility for the Tanzanian cohort was based on HIV seronegativity.

### Comparing the Diary Study cohorts to each other

There were substantial differences between the 2 diary study cohorts related to employment and sexual behaviour at baseline. The majority of the participants in the Ugandan Diary Study were FSW (61%) compared with Tanzania where only bar-workers and other facility-based workers were enrolled. Ugandan participants were less likely to be married, had more lifetime partners, a younger age at first sex, more reported consistent condom use, and more reported transactional sex. Most Ugandan (66%), and over a quarter of Tanzanian (26%), participants could not remember their total lifetime partners, possibly because the number of partners was very high.

### Intravaginal cleansing: per-woman analysis

In Tanzania and Uganda, 96.3% and 100% of women respectively reported cleansing at least once in the diary (Table 2). Most women cleansed several times a day (median cleansing acts/day in Tanzania  = 4.0 and in Uganda  = 4.7). A large proportion used a commercial product at least once, 59% in Tanzania and 77% in Uganda. In both Diary Study cohorts, about half of the women who reported use of commercial products to cleanse used them more than 75% of times they cleansed. In contrast, far fewer participants used a traditional product, and almost all women who reported use of traditional products used them less than 25% of the times they cleansed. In both countries, IDI revealed the commercial products used for cleansing were usually soap, although in Uganda aerated drinks (e.g. Coca-Cola) and salt were also reported. Tanzanian participants did not report the use of traditional products to cleanse; in Uganda, participants reported the use of herbs.

**Table 2:** Summary of cleansing data reported in the diaries over 42 days by women who had complete data in the Diary Study in Tanzania and Uganda, per woman analysis.

	Tanzanian Diaries(N = 82)	Ugandan Diaries(N = 99)	Comparison(p-value)^a^
**Frequency**			
Number of women who report cleansing ever	79 (96.3%)	99 (100.0%)	p = 0.06
Mean cleansing acts per day^b^	3.7	4.5	
Median (IQR) cleansing acts per day^b^	4.0 (3.2 to 4.6)	4.7 (4.2 to 5.0)	p<0.01
Mean frequency of cleansing per day^b^	(n = 82)	(n = 99)	p<0.01
<0.50	3 (3.7%)	0 (0%)	
0.50 to 1.49	3 (3.7%)	0 (0%)	
1.50 to 2.49	6 (7.3%)	0 (0%)	
2.50 to 3.49	13 (15.9%)	5 (5.1%)	
3.50 to 4.49	32 (39.0%)	34 (34.3%)	
>4.50	25 (30.5%)	60 (60.6%)	
**Substance**	(n = 82)	(n = 99)	
Overall substance use			p<0.01
Water only always	28 (34.2%)	19 (19.2%)	
Commercial product at least once, but never traditional^c^	46 (56.1%)	44 (44.4%)	
Traditional product at least once, but never commercial^d^	3 (3.7%)	4 (4.0%)	
Both commercial and traditional products at least once, separately	1 (1.2%)	18 (18.2%)	
Both commercial and traditional products at least once, combined	1 (1.2%)	14 (14.1%)	
Does not cleanse	3 (3.7%)	0 (0%)	
Frequency of using a commercial product as proportion of times cleansed^e^	(n = 48)	(n = 76)	p = 0.38
<25%	12 (25%)	24 (31.6%)	
25 to 49%	4 (8.3%)	4 (5.3%)	
50 to 74%	6 (12.5%)	11 (14.5%)	
75% +	26 (54.2%)	37 (48.7%)	
Frequency of using a traditional product as proportion of times cleansed^f^	(n = 5)	(n = 36)	p = 0.12
<25%	5 (100%)	33 (91.7%)	
25 to 49%	0 (0%)	3 (8.3%)	
50 to 74%	0 (0%)	0 (0%)	
75% +	0 (0%)	0 (0%)	
**Application**	(n = 82)	(n = 99)	
Application method			p<0.01
Fingers only always	46 (56.1%)	36 (36.4%)	
Cloth at least once, but never other	20 (24.4%)	19 (19.2%)	
Other^g^ at least once, but never cloth	6 (7.3%)	14 (14.1%)	
Both cloth and other at least once, separately	2 (2.4%)	21 (21.2%)	
Both cloth and other at least once, combined	5 (6.1%)	9 (9.1%)	
Does not cleanse	3 (3.7%)	0 (0%)	
Frequency of using cloth, as proportion of times cleansed	(n = 27)	(n = 49)	p = 0.45
<25%	17 (63.0%)	32 (65.3%)	
25 to 49%	0 (0%)	5 (10.2%)	
50 to 74%	4 (14.8%)	3 (6.1%)	
75% +	6 (22.2%)	9 (18.4%)	
Frequency of using another applicator, as proportion of times cleansed	(n = 13)	(n = 44)	p = 1.00
<25%	0 (0%)	0 (0%)	
25 to 49%	0 (0%)	0 (0%)	
50 to 74%	0 (0%)	0 (0%)	
75% +	13 (100%)	44 (100%)	
**Cleansing related to sexual intercourse**			
Number of women who had sex at least once during the study	69 (84.2%)	96 (97.0%)	p<0.01
Mean number of sex acts per day^h^	0.43	1.67	
Median (IQR) of sex acts per day^h^	0.29 (0.19 to 0.60)	1.37 (0.91 to 2.41)	p<0.01
Frequency of sex-related cleansing, as a proportion of the woman’s total sex acts^i^	(n = 57)	(n = 71)	p<0.01
Never	3 (5.3%)	3 (4.2%)	
<25%	1 (1.8%)	2 (2.8%)	
25 to 49%	3 (5.3%)	9 (12.7%)	
50 to 74%	8 (14.0%)	27 (38.0%)	
75% +	42 (73.7%)	30 (42.3%)	
Mean cleansing acts per day when NO sex was reported	3.78	4.46	
Mean cleansing acts per day when sex was reported	3.99	4.56	
p-values for the difference between distributions^j^	p = 0.01	p = 0.08	
**Cleansing related to menses**			
Frequency of menstruation			p = 0.13
2+ menstrual periods during study	31 (37.8%)	51 (51.5%)	
One menstrual period during study	39 (47.6%)	33 (33.3%)	
No menstrual periods during study	12 (14.6%)	15 (15.2%)	
Cleansing related to menstruation	(n = 70)	(n = 84)	
Mean cleansing acts per day when there was NO menstruation	3.68	4.50	
Mean cleansing act per day when there was menstruation	3.98	4.67	
p-values for the difference between distributions^j^	p<0.01	p<0.01	
Sex during menstruation	(N = 70)	(N = 84)	
Participants who reported sex at least once during a day of menstruation	8 (11.4%)	34 (40.5%)	p = 0.01
**Vaginal cleansing related to vaginal discomfort^k^**			
Frequency			
Participants who noted vaginal discomfort at least once	40 (48.8%)	60 (60.6%)	0.13
Cleansing related to vaginal discomfort	(n = 40)	(n = 60)	
Mean cleansing acts per day when there was NO vaginal discomfort	3.69	4.54	
Mean cleansing acts per day when there was vaginal discomfort	3.89	4.62	
p-values for the difference between distributions^j^	p = 0.39	p = 0.08	

**Legend:** a  =  Wilcoxon rank-sum for non-parametric data; b  =  Calculation was for all participants including those not reporting cleansing. Up to four cleansing acts could be counted. If a participant ticked the box stating she had cleansed more than four times on that day, we assumed that she had cleansed five times; c  =  Commercial products for cleansing: In In-depth Interviews (IDI), participants reported the use of soap in Tanzania and soap and aerated drinks in Uganda; d =  Traditional products for cleansing: In the IDI, Tanzanian participants did not report the use of traditional products, but in Uganda the use of herbs was reported; e  =  Restricted to those who reported using a commercial substance; f  =  Restricted to those who reported using a traditional substance; g  =  Other applicator used for cleansing: In IDI, participants reported the use of toilet paper in Tanzania and Uganda; h  =  Restricted to those who reported having sex; i  = Among women who reported cleansing at least once and had sex at least once, restricted to the days when they reported cleansing ≤4 times so that all sex-related cleansing could be captured by the diary; j  =  Wilcoxon signed-rank test for paired data; k  =  Vaginal discomfort was defined as vaginal irritation, pain or itching; an abnormal discharge; or abnormal smell.

Over half the participants in Tanzania (56.1%) and 36.4% in Uganda used only their fingers to apply the product every time they cleansed. Around one third of women in Tanzania (32.9%) and a half in Uganda (49.5%) reported using cloths on at least one occasion, either alone or in combination with another applicator. Most cloth-users used cloth less than 25% of the total times they cleansed. In contrast, of the women who used another applicator, all of them used it more than 75% of total time cleansed. In the IDIs, participants in both cohorts reported that the ‘other’ applicator was toilet paper.

Overall, 84.2% of Tanzanian participants and 97.0% of Ugandan participants reported at least one sex act during the study (median 0.3 sex acts/day and 1.4 sex acts/day, respectively). In both Diary Study cohorts, among the women who reported sex, there was evidence that participants cleansed more frequently on days when they reported sex than on days when no sex was reported (p = 0.01 for Tanzania; p = 0.08 for Uganda, Table 2).

Participants reported cleansing ≤4 times a day on 69% of diary days in Tanzania, and 37% of diary days in Uganda. In an analysis restricted to these days, 42 (73.7%) of women in Tanzania and 30 (42.3%) in Uganda reported cleansing, either before or after, accompanying most (≥75%) of their sex acts.

During the six weeks of reporting, the majority of women reported at least one menstrual period (85.4% in Tanzania and 84.8% in Uganda). Among those women, participants in both cohorts cleansed more frequently on days when they were menstruating (p<0.01, Table 2). Around 11% of Tanzania participants reported sex during menses compared with 40% of Ugandan participants.

Around half (48.8%) of the Tanzanian participants and 60.6% of the Ugandan participants reported vaginal discomfort at least once during the six weeks. In Tanzania, there was no evidence of a difference in the frequency of cleansing on days with vaginal discomfort (p = 0.39, Table 2); however, in Uganda there was some evidence that women cleansed more frequently on days when they reported vaginal discomfort (p = 0.08).

### Intravaginal insertion: per-woman analysis

Insertion practices were very different between the Diary Study cohorts (Table 3). In Tanzania, only eight (9.8%) participants reported insertion during the six weeks. Of the Tanzanian participants who inserted, the median (IQR) insertion acts during the study was 4.5 (2.0 to 29.0). One woman used only commercial products, four used only traditional products, and three used both commercial and traditional products. Three women always used fingers to apply the insertion substance intravaginally, but the other five used cloth or another type of applicator. There was no evidence of a difference in the frequency of insertion on days when sex was reported and when sex was not reported (p = 0.77). In the IDI, only traditional products were reported in Tanzania: herbs, ghee, snuff, and lemons, and “other” applicator was reported as toilet paper or medical applicator.

**Table 3:** Summary of insertion data reported in the diaries over 42 days by woman who had complete data in the Diary Study in Tanzania and Uganda, per woman analysis.

	Tanzanian Diaries(N = 82)	Ugandan Diaries(N = 99)	Comparison(p-value)^a^
**Frequency**			
Number of women who report insertion ever	8 (9.8%)	45 (45.5%)	p<0.01
Mean insertion acts during the six weeks of the study^b^	20.6	40.8	
Median (IQR) insertion acts in the study^b^	4.5 (2.0 to 29.0)	19.0 (9.0 to 67.0)	p = 0.04
Frequency of insertion acts in the study^b^			p = 0.03
1 to 5 times	5 (62.5%)	7 (15.6%)	
6 to 10 times	1 (12.5%)	7 (15.6%)	
11 to 20 times	0 (0.0%)	10 (22.2%)	
> 20 times	2 (25.0%)	21 (46.7%)	
**Substance**			
Overall substance use	(n = 8)	(n = 45)	p = 0.71
Commercial product only always^c^	1 (12.5%)	9 (20.0%)	
Traditional product only always^d^	4 (50.0%)	7 (15.6%)	
Both commercial and traditional products at least once, separately	1 (12.5%)	23 (51.1%)	
Both commercial and traditional products at least once, combined	2 (25.0%)	6 (13.3%)	
**Application**			
Application method	(n = 8)	(n = 45)	p = 0.98
Fingers only always	3 (37.5%)	16 (35.6%)	
Cloth at least once but never other	2 (25.0%)	7 (15.6%)	
Other^e^ at least once but never cloth	1 (12.5%)	12 (26.7%)	
Both cloth & Other at least once, separately	0	9 (20.0%)	
Both cloth and Other at least once, combined	2 (25.0%)	1 (2.2%)	
**Insertion related to sexual intercourse**			
Frequency of sex-related insertion, as a proportion of the woman’s total sex acts^ f^	(n = 6)	(n = 43)	p = 0.02
Never	3 (50.0%)	11 (25.6%)	
<25%	3 (50.0%)	23 (53.5%)	
25 to 49%	0	7 (16.3%)	
50 to 74%	0	2 (4.7%)	
75% +	0	0	
	(n = 8)	(n = 45)	
Mean insertion acts per day when NO sex was reported	0.27	0.38	
Mean insertion acts per day when sex was reported	0.21	0.91	
p-values for the difference between distributions^g^	p = 0.69	p<0.01	

**Legend:** a  =  Wilcoxon rank-sum for non-parametric data; b  =  This was restricted to those who reported insertion; c  =  Commercial products for insertion: In In-depth Interviews (IDI), no participant reported the use of a commercial product in Tanzania, and in Ugandan commercial products reported were soda (e.g. Coca-Cola), medication for vaginal infections, laundry detergent, petroleum-based jelly, and beer; d  =  Traditional products for insertion: In IDI, participants reported the use of herbs, ghee, snuff, and lemons in Tanzania, and herbs and honey in Uganda; e  =  Other applicator used for insertion: In IDI, participants reported the use of toilet paper and applicator (e.g. to insert medication) in Tanzania, and toilet paper in Uganda; f  =  Among women who reported insertion at least once and had sex at least once, restricted to the days when they reported inserting ≤2 times so that all sex-related insertion could be captured by the diary; g  =  Wilcoxon signed-rank test for paired data.

In contrast, in Uganda, insertion practices were more common with half of the women (49.5%) reporting insertion at least once during the six weeks of the study. Of the Ugandan participants who inserted, the median (IQR) insertion acts during the study was 19.0 (9.0 to 67.0) or about 3 times per week. One-fifth of participants who inserted used only commercial products at every insertion, and 15.6% used only traditional products. Many (36.7%) women used fingers only every time they applied the insertion substance intravaginally, but the remainder used cloth or another type of applicator at least once. There was strong evidence that insertion was more frequent on days when sex was reported (p<0.01). IDI revealed that commercial products were aerated drinks, medications for vaginal infections, petroleum-based jelly, laundry detergent, and beer; traditional products were herbs and honey; and “other” applicators were applicators (i.e. to insert vaginal medication) and toilet paper.

Participants reported inserting ≤2 times a day on 99% of diary days in Tanzania, and 91% of diary days in Uganda. In an analysis restricted to those days, 50% of women in Tanzania who inserted never reported the practice in relation to sex; among the remaining half, insertion accompanied <25% of sex acts. In contrast, in Uganda, most women who inserted reported the practice in relation to sex on at least some occasions.

### Cleansing and Insertion: per act analysis

In both Diary Study cohorts, water alone was used for over half of cleansing acts (62.4% in Tanzania and 52.3% in Uganda, Table 4); a commercial product combined with water was used at most other times. Fingers alone were used to apply the cleansing substance intravaginally in over 80% of acts, with a small percentage of acts using cloths, either alone or with fingers (11.7% in Tanzania and 14.6% in Uganda). In Tanzania, only 15% of all cleansing acts were related to sex, with 55% of those being after sex. In Uganda, since women reported sex more frequently than in Tanzania, a higher proportion of all cleansing acts were related to sex (41.3%); around half (47%) the sex-related cleansing acts were after sex. In both Diary Study cohorts, when cleansing acts were reported after sex, there was a lower percentage of condom use than for cleansing acts reported before sex. In a sensitivity analysis restricted to the days when women only cleansed before sex or after sex, but not both, we found a similar lower prevalence of condom use associated with cleansing after sex (data not shown). In the analysis restricted to the days on which all sex-related cleansing could be captured by the diary, 75% of sex acts in Tanzania were accompanied with cleansing after sex, and 70% with cleansing before. In contrast, in Uganda, the proportion of sex acts accompanied by cleansing was lower: 50% with cleansing after sex and 48% with cleansing before.

**Table 4:** Summary of cleansing and insertion data reported in the diaries over 42 days by act.

	Tanzanian Diaries(N = 82)	Ugandan Diaries(N = 99)	Comparison(p-value)^a^
**CLEANSING** (Diary rows C1 to C4)			
**Frequency**			
Total number of cleansing acts in the study	11,649	16,145	
Overall substance by total number of acts			p = <0.01
Water only	7,272 (62.4%)	8,441 (52.3%)	
Commercial^b^ and water	4,325 (37.1%)	6,980 (43.2%)	
Traditional^c^ and water	18 (0.2%)	423 (2.6%)	
Commercial only	3 (0.0%)	146 (0.9%)	
Traditional only	0 (0.0%)	16 (0.1%)	
Commercial, traditional and water	3 (0.0%)	40 (0.3%)	
Commercial and traditional	0 (0.0%)	2 (0.0%)	
Missing^d^	28 (0.2%)	97 (0.6%)	
**Application**			
Overall method by total number of acts			<0.01
Fingers only	10,218 (87.7%)	13,415 (83.8%)	
Cloth only	471 (4.0%)	412 (2.6%)	
Other only^e^	2 (0.0%)	48 (0.3%)	
Cloth and finger	902 (7.7%)	1,919 (12.0%)	
Other and finger	33 (0.3%)	202 (1.3%)	
Cloth, other and finger	7 (0.1%)	6 (0.0%)	
Cloth, other	16 (0.2%)	16 (0.1%)	
Missing^d^	0 (0%)	128 (0.8%)	
**Cleansing related to sexual intercourse & condom use**			
Total sex-related cleansing acts, as proportion of all cleansing	1743 (15.0%)	6665 (41.3%)	
Of the total sex-related cleansing acts, how many were before sex	782 (44.9%)	3506 (52.6%)	
Of the total cleansing acts before sex, how many used a condom	370 (47.3%)	2,841 (81.0%)	<0.01
Of the total sex-related cleansing acts, how many were after sex	961 (55.1%)	3159 (47.4%)	
Of the total cleansing acts after sex, how many used a condom	208 (21.6%)	710 (22.5%)	0.59
Total number of sex acts during the study	1254	6749	
Total number of sex acts on days when cleansed ≤4 times	864	2137	
Of the total sex acts, how many were associated with before sex cleansing	527 (70.0%)	1033 (48.3%)	
Of the total sex acts, how many were associated with after sex cleansing	646 (74.8%)	1077 (50.4%)	
**▪INSERTION** (Diary rows I1 to I2)			
**▪Frequency**			
Total number of insertion acts in the study	155	1,444	
Overall substance by total number of acts			<0.01
Commercial product only^f^	3 (1.9%)	756 (52.4%)	
Traditional product only^g^	148 (95.5%)	680 (47.1%)	
Commercial and traditional product together	4 (2.6%)	8 (1.0%)	
**Application**			
Overall method by total number of acts			<0.01
Finger only	144 (87.8%)	1,071 (74.7%)	
Cloth only	0 (0.0%)	66 (4.6%)	
Other only^h^	0 (0.0%)	99 (6.9%)	
Cloth and finger	6 (3.9%)	156 (10.9%)	
Other and finger	1 (0.7%)	40 (2.8%)	
Cloth and other	0 (0.0%)	1 (0.1%)	
Cloth, finger and other	4 (2.6%)	0 (0.0%)	
Missing^d^	0 (0%)	11 (0.8%)	
**Insertion related to sexual intercourse & condom use**			
Total sex-related insertion acts, as proportion of all insertion	7 (4.5%)	978 (67.7%)	<0.01
Of the total sex-related insertion acts, how many were before sex	6 (85.7%)	590 (60.3%)	<0.01
Of the total insertion acts before sex, how many used a condom	3 (50.0%)	461 (78.1%)	0.03
Of the total sex-related insertion acts, how many were after sex	1 (14.3%)	388 (39.7%)	<0.01
Of the total insertion acts after sex, how many used a condom	0 (0%)	80 (20.6%)	0.61

**Legend:** a  =  Wilcoxon rank-sum for non-parametric data;; b  =  Commercial products for cleansing: In In-depth Interviews (IDI), participants reported the use of soap in Tanzania, and soap, soda (e.g. Coca-Cola), salt, and laundry detergent in Uganda; c  =  Traditional products for cleansing: In the IDI, Tanzanian participants did not report the use of traditional products, but in Uganda the use of herbs was reported; d = data was missing for substance or applicator, but cleansing or insertion was ticked; e  =  Other applicator used for cleansing: In IDI, participants reported the use of toilet paper in both Tanzania and Uganda; f  =  Commercial products for insertion: In In-depth Interviews (IDI), no participants reported the use of a commercial product in Tanzania, and in Uganda, participants reported the use of soda (e.g. Coca-Cola), medication for vaginal infections, laundry detergent, petroleum-based jelly, and beer; g  =  Traditional products for insertion: In IDI, participants reported the use of herbs, ghee, snuff and lemon in Tanzania, and herbs and honey in Uganda; h  =  Other applicator used for insertion: In IDI, participants reported the use of toilet paper in Tanzania, applicator (e.g. to insert medication), and toilet paper in Uganda.

In Tanzania, almost all the insertion acts were with a traditional product, and in Uganda about half the insertion acts were with a commercial product alone, and the other half were with a traditional product alone; very few combined traditional and commercial products. In both cohorts, most women used fingers only, the remainder using cloth alone or in combination with fingers. In Tanzania, only 7 (4.5%) insertion acts were related to sex. In contrast, in Uganda, 67.7% of all insertion acts were related to sex. In Uganda, when insertion acts were reported after sex, there was a lower percentage of condom use than for insertion acts reported before sex. The proportion of all sex acts accompanied by insertion was low in both cohorts (0.5% in Tanzania and 5% in Uganda).

## Discussion

IVP are highly prevalent among women at increased risk for HIV infection. Several recent publications have attempted to quantify this increased risk through meta-analyses [1], [2]; however, this may be problematic if IVP are heterogeneous between study populations. Our study compared two populations of women at high risk for HIV in East Africa using a vaginal practice diary, and revealed similarities between the study populations and several important differences related to sexual behaviour. Intravaginal cleansing was highly normative among the Tanzanian and Ugandan participants. Most women used water only or water and a commercial product (e.g. soap) for cleansing, and most applied the substances with only their fingers. In both countries, intravaginal insertion was relatively less common than cleansing. However, in Uganda there was more frequent cleansing, more use of traditional products for cleansing, more insertion, more sex acts, and more insertion related to sex acts. Our study used the same data collection tools and definition of IVP to obtain data and has demonstrated important heterogeneity of IVP between two populations.

Our findings strongly suggest that the differences in IVP frequency were related to frequency of sex. Although in the per-act analysis, a higher proportion of all sex acts in Tanzania participants were accompanied by cleansing, the Uganda participants had a higher frequency of cleansing overall, with 41% of cleansing acts being related to sex. The difference in insertion practices was particularly dramatic, with two-thirds of insertion acts in Uganda being related to sex, compared with <5% in Tanzania. Differences in sexual behaviour are likely to reflect the differences in the cohorts highlighted in Table 1: the Ugandan cohort enrolled FSW and had a higher proportion of women reporting transactional sex and more life-time partners. Female facility workers have been shown to supplement their income with transactional sex and have high HIV prevalence; however, previous research in Tanzania has found that bar workers and other facility workers have fewer sexual encounters than FSW in the same sexual network [21].

Our results show that a large proportion of cleansing acts are not related to sex, but most sex acts are accompanied by IVP. In other words, women are cleansing for a variety of reasons, including, but not limited to, cleansing before or after sex. This is evident from a much larger mean number of cleansing acts than mean number of sex acts per day. However, our ability to fully describe sex-related IVP was limited by the diary design, since it could only capture a maximum of 4 cleansing and 2 insertion acts per day. Nevertheless, both Diary Study cohorts reported cleansing more frequently on the days they had sex, and our findings suggest that a large proportion of participants were cleansing before or after the majority of their sex acts. This would be a concern if cleansing increases susceptibility to sexually transmitted or reproductive tract infections, and if products commonly used for cleansing interfere with an efficacious topical microbicide.

Increased cleansing was reported concurrently with sexual activity and menstruation in both groups. Vaginal discomfort was also reported together with increased cleansing, although the evidence is weaker in the Tanzanian cohort, perhaps due to the low number of women reporting vaginal discomfort. Qualitative research from this study revealed that many women are initiated to intravaginal cleansing at the time of their first menses, and a main purpose is to remove “dirt” (Swahili -*Uchafu and* Luganda *– Obukyafu,* which refers to body fluids) to maintain health and avoid infection.

Interestingly, reported consistent condom use was higher in the Tanzania Diary Study than the main cohort, and was higher still in the Ugandan cohorts than in Tanzania. This parallels a higher proportion of women reporting transactional sex in the Tanzanian Diary Study than in the main cohort, and in the Ugandan cohorts than in the Tanzanian cohorts. This may reflect a higher rate of consistent condom use during transactional sex. Additionally, in both cohorts, there was a lower prevalence of condom use with the co-occurrence of cleansing after sex, than with before-sex cleansing. This finding suggests that participants may be cleansing after sex as a response to the lack of condom use, for example, because of concerns about hygiene, infection or pregnancy. Our qualitative research suggested that some women may be encouraged or even forced by clients to not use condoms, and thus women may cleanse after sex in an effort to protect themselves from infections or pregnancy.

Our findings from the per-woman and per-act analyses revealed important limitations of using FTFI to gather IVP data. In both countries, the majority of Diary Study participants reported using a commercial product at least once. This finding is supported by FTFI data from main cohort baseline data in Tanzania and Uganda [19], [22], and in a recently published individual person data analysis that pooled IVP data from FTFI from eight studies in sub-Saharan Africa [1]. However, the per-act analysis shows that the majority of cleansing acts were with water only. This highlights the limitations of FTFI when asking participants if they have “ever used” a substance, since this may mask the most common substance used, as well as potentially misclassify participants who rarely used a product other than water.

A major strength of our study was the use of a self-completed diary which is likely to decrease social desirability bias, and can be completed near real-time to decrease recall bias. In addition, we were able to capture the co-occurrence of menstruation, vaginal irritation (i.e. vaginal itching, burning, discharge or smell) and sex, which are likely to modify daily IVP frequency. The diaries were designed to be locally appropriate, and there was good retention in both the study sites.

There were several limitations of our study. The diary design allowed a maximum of four cleansing acts per day and two insertion acts per to be described, while a substantial proportion of participants in both sites reported cleansing more than four times a day. Furthermore, we could not assess whether IVP before and after sex related to the same sex act, or separate ones. In addition, the days when women cleansed ≤4 times or inserted ≤2 times may not be typical; therefore our estimates of the amount of sex-related cleansing as a proportion of all sex acts may be biased, especially in Uganda where women cleanse >4 times on 63% of days. Lastly, although participants were instructed to complete the diary daily, it is possible that participants neglected to do so; however, at worst, diaries would have been completed once a week before collection by the research assistant.

In conclusion, several important findings related to IVP were revealed in the Diary Study. While intravaginal cleansing was commonly practiced in both cohorts, there was higher frequency of cleansing, sex-related cleansing, and insertion in Uganda, suggesting differences in IVP are due to sexual behaviour related to employment (FSW vs. facility workers). These differences warrant caution when combining effect estimates of IVP in meta-analyses, and may require different approaches to interventions targeting IVP. In addition, transient conditions such as menstruation and vaginal discomfort are likely to increase the frequency of cleansing acts. Lastly, while many participants used a product other than water at least once during the study, the majority of cleansing acts were with water only, highlighting possible misclassification in “ever used” survey questions. Survey data may be improved by documenting the frequency of products used. The comparison of IVP between two study populations affirms that IVP is a complex behaviour; more research is needed to understand the risks related to IVP, microbicide use, and sexually transmitted and reproductive tract infections, including HIV.
